# Anemia in an ethnic minority group in lower northern Thailand: A community-based study investigating the prevalence in relation to inherited hemoglobin disorders and iron deficiency

**DOI:** 10.1371/journal.pone.0287527

**Published:** 2023-06-23

**Authors:** Thinzar Win Pyae, Kanokwan Sanchaisuriya, Surasak Athikamanon, Pattara Sanchaisuriya, Hataichanok Srivorakun, Attawut Chaibunruang, Supan Fucharoen

**Affiliations:** 1 Graduate School, Khon Kaen University, Khon Kaen, Thailand; 2 Centre for Research and Development of Medical Diagnostic Laboratories (CMDL), Faculty of Associated Medical Sciences, Khon Kaen University, Khon Kaen, Thailand; 3 Boromrajonani College of Nursing Chai Nat, Chai Nat, Thailand; 4 Foundation of Community System Research and Development Institute, Bangkok, Thailand; 5 Faculty of Public Health, Khon Kaen University, Khon Kaen, Thailand; Menzies School of Health Research, AUSTRALIA

## Abstract

**Background:**

Anemia is a globally well-known major public health problem. In Southeast Asia where there is ethnic diversity, both iron deficiency (ID) and inherited hemoglobin disorders (IHDs) are prevalent and are considered to be the major factors contributing to anemia. However, little is known about the anemia burden among the ethnic minorities. In this study, we determine the burden of anemia, in relation to ID and IHDs, among the Karen ethnic minorities living in the rural area of lower northern Thailand.

**Methods:**

A cross-sectional community-based study was conducted at Ban Rai district, Uthai Thani province. Study participants included 337 Karen people aged over 18 years. Socio-economic and health-related information were obtained through interviews and recorded by local health staff. Anemia, IHDs and ID were diagnosed according to standard laboratory methods. Multivariate logistic regression analysis was applied to identify risk factors of moderate-to-severe anemia.

**Results:**

The prevalence of overall anemia was 27.9% (95% CI = 23.2–33.0). Mild and moderate anemia were detected in 18.7% (95% CI = 14.7–23.3) and 8.9% (95% CI = 6.1–12.5) respectively. Severe anemia was found in one case (0.3%). Various forms of IHDs were identified in 166 participants, constituting 49.3% (95% CI = 43.8–54.7). The most common form of IHDs was α^+^-thalassemia (32.9%), followed by β-thalassemia (12.2%), α^0^-thalassemia (4.2%), hemoglobin E (3.9%), and hemoglobin Constant Spring (0.9%). Among 308 participants who were investigated for ID, the prevalence was discovered to be 6.8% (95% CI = 4.3–10.2). Analysis of risk factors of moderate-to-severe anemia revealed that individuals with ID, β-thalassemia and age > 65 years were at high risk with adjusted odds ratio of 17 (95% CI = 3.8–75.2), 6.2 (95% CI = 1.4–27.8) and 8.1 (95% CI = 1.6–40.4) respectively.

**Conclusions:**

Anemia among the Karen is of public health significance; and IHDs are the major contributing factors. Because of the high risk of developing moderate-to-severe anemia, special attention should be paid to individuals affected with ID, β-thalassemia and the elderly. Public awareness of the health burden of severe thalassemia syndromes should also be campaigned.

## Background

Anemia, the most common hematologic abnormality, remains a global public health burden, especially in low and low-middle income countries [1]. It is characterized by the low production of hemoglobin (Hb) and/or red blood cells (RBC). In individuals aged over 15 years, anemia is defined by Hb < 130 g/L for males and 120 g/L for females [2]. Depending on Hb levels, anemia can be classified as mild (Hb = 110–119 g/L for females and 110–129 g/L for males), moderate (Hb = 80–109 g/L), and severe (Hb < 80 g/L). Due to the decreased oxygen-carrying capacity of Hb, anemic individuals may experience unpleasant symptoms such as weakness, dizziness, fatigue, shortness of breath, chest pain and palpitations, and this can lead to reduced work productivity. In severe cases, risk of morbidity and mortality is increased, especially in children and pregnant women [2, 3].

Although there are various causes that give rise to anemia, iron deficiency (ID) is considered as the main cause. In certain populations, inherited Hb disorders (IHDs) have been reported to be the major determinants of anemia [4–6]. At the molecular level, IHDs are broadly classified into thalassemia and structural Hb variants. While thalassemia results from a decreased or absent globin chain production, a structural Hb variant results from an alteration of the globin chain structure [7]. Heterozygous states for IHDs usually have no clinical symptoms; however, mild anemia might be detected due to the low Hb production within RBCs. The prevalence of IHDs is particularly high in tropical regions including Southeast Asia, where the prevalence of ID is presumably high. Common forms of IHDs in this region include α^0^-thalassemia (α^0^-thal), α^+^-thalassemia (α^+^-thal), β-thalassemia (β-thal), Hb E and Hb Constant Spring (Hb CS). Depending on ethnic background, community-based surveys in this region have shown varying frequencies, ranging from 2–16% for α^0^-thal and 10–35% for α^+^-thal, 0.5–10% for β-thal, 2–60% for Hb E and 2–25% for Hb CS [8–15]. Interaction of these IHD genes can lead to thalassemia syndromes with varying anemia severities.

In Thailand, there are more than 30 ethnic minority groups in which the Karen is the largest one. Although the origin of the Karen people is uncertain, it is believed that they migrated from the Gobi Desert, Mongolia or Tibet, and settled in the Ayeyarwady (also called Irrawaddee) Delta and in the hills along the Salween River in Myanmar and Thailand [16]. The Karen minorities are divided into 4 subgroups, including Sgaw, Pwo, Pa’O, and Kayah. Each sub-group has a distinct language and culture. Most of the Karen in Thailand live in the areas close to the Myanmar border across 20 provinces in the north and north-west. Traditionally, the Karen people live together in mountainous highlands or in an open space in a forest. Due to their poverty and the inaccessibility of healthcare services, minorities are prone to anemia and other health problems.

This study aimed to determine the burden of anemia among the Karen ethnic minorities residing in rural areas of Ban Rai district, Uthai Thani province, Thailand. We hypothesized that IHDs and ID among the study population might be high. Therefore, we also determined the contributions of IHDs and ID to anemia prevalence stratified by severity and risk factors for the development of moderate-to-severe anemia.

### Materials and methods

#### Study design and participants

A community-based study was conducted at Ban Rai district, Uthai Thani province, Thailand. The study population included the Karen ethnic minorities residing in 4 villages of the district. The Karen minorities settled in this area belong to the “Pwo” subgroup and called themselves "Phlong" or "Kêphlong." They live together in an open space of the forest and conserve the culture of farming. To recruit participants, convenience sampling was applied. A sample size was calculated using a formula for infinite population proportion. With an expected anemia prevalence of 25% and 95% confidence interval at 5% marginal error, at least 298 samples were required. Only apparently heathy males and non-pregnant females aged over 18 years were included. The study was approved by the Ethics Committee of Khon Kaen University (HE642267). On a voluntary basis, a total of 337 Karen people were enrolled. Written informed consent was obtained from all participants.

Field work was carried out between March and April 2022. Prior to data collection, the project was introduced to the Karen people via community loudspeaker. Details of the project (including the purpose, procedures, anticipated outcomes, benefit and risk to participants) were made known to all eligible participants by the village health volunteers. Socio-demographic and health-related information (including age, sex, weight, height, educational level, occupation, underlying disease, major blood loss, iron supplementation, and a history of thalassemia disease in the family) were obtained through interviews and recorded by the local health staff. All participants were free to decline to answer any question or withdraw from the project at any time. On an appointment date, a venous blood sample was taken from each participant, 2 mL as K3-EDTA blood, and 3 mL as clotted blood. All blood samples were kept in a cool box and sent to a community hospital where a complete blood count (CBC) and serum separation were performed. The residual EDTA blood and serum samples were stored at 2–6°C and transported to the Centre for Research and Development of Medical Diagnosis Laboratories (CMDL), Khon Kaen University, Thailand, for further laboratory investigations. All data were kept confidential. To protect participant confidentiality and validate the accuracy of data, only the principal investigator and the project administrator had access to information that could identify individual participants during or after data collection.

### Laboratory investigations

Within 4 hours of blood collection, CBC was determined using an automated blood cell counter (Sysmex KX-21, Sysmex Co, Kobe, Japan). Anemia was defined according to the WHO criteria, i.e., Hb < 120 g/L for females and Hb < 130 g/L for males. Anemia severity was classified into 3 groups as follows: mild (Hb = 110–119 g/L for females and 110–129 g/L for males), moderate (Hb = 80–109 g/L), and severe anemia (Hb < 80 g/L) [2]. To identify individuals with IHDs, Hb E was initially screened for, using the KKU-DCIP-Clear reagent kit (PCL Holdings, Bangkok, Thailand). Hb separation was done at alkaline pH using the Titan III cellulose acetate (Helena Laboratories, Texas, USA). Samples showing normal Hb type (A_2_A) with MCV < 80 fL and/or mean corpuscular hemoglobin (MCH) < 27 pg were subject to further quantitation of Hb A_2_ level using an automated capillary electrophoresis (Capillarys II; Sebia, Leisse, France). An individual with Hb A_2_ > 4% was diagnosed as being a β-thal carrier [17]. Genotyping of IHDs was performed using PCR-based technologies. Six α-thal mutations common in Southeast Asia, including SEA and THAI deletion α^0^-thal, -3.7 and -4.2 kb deletion α^+^-thal, Hb CS and Hb Pakse′ were investigated using multiplex gap-PCR and allele specific PCR (ASPCR), as described previously [18–20]. All β-thal carriers were investigated for β-globin gene mutations using the previously described methods [21].

To assess the iron status of each individual, serum ferritin (SF) was measured using the chemiluminescent microparticle immunoassay (CMIA) (Abbott; ARCHITECT i2000SR Immunoassay, IL, USA). C-reactive protein (CRP), a marker of inflammation, was determined using the CRP Latex Test Kit (Plasmatec Co., Dorset, UK). An individual with negative CRP and SF level < 15 ng/mL (33.7 pmol/L) was diagnosed as having ID. For those with positive CRP, the diagnosis of ID was based on SF < 70 ng/mL (157.3 pmol/L) [22].

### Statistical analysis

**Fig 1:**
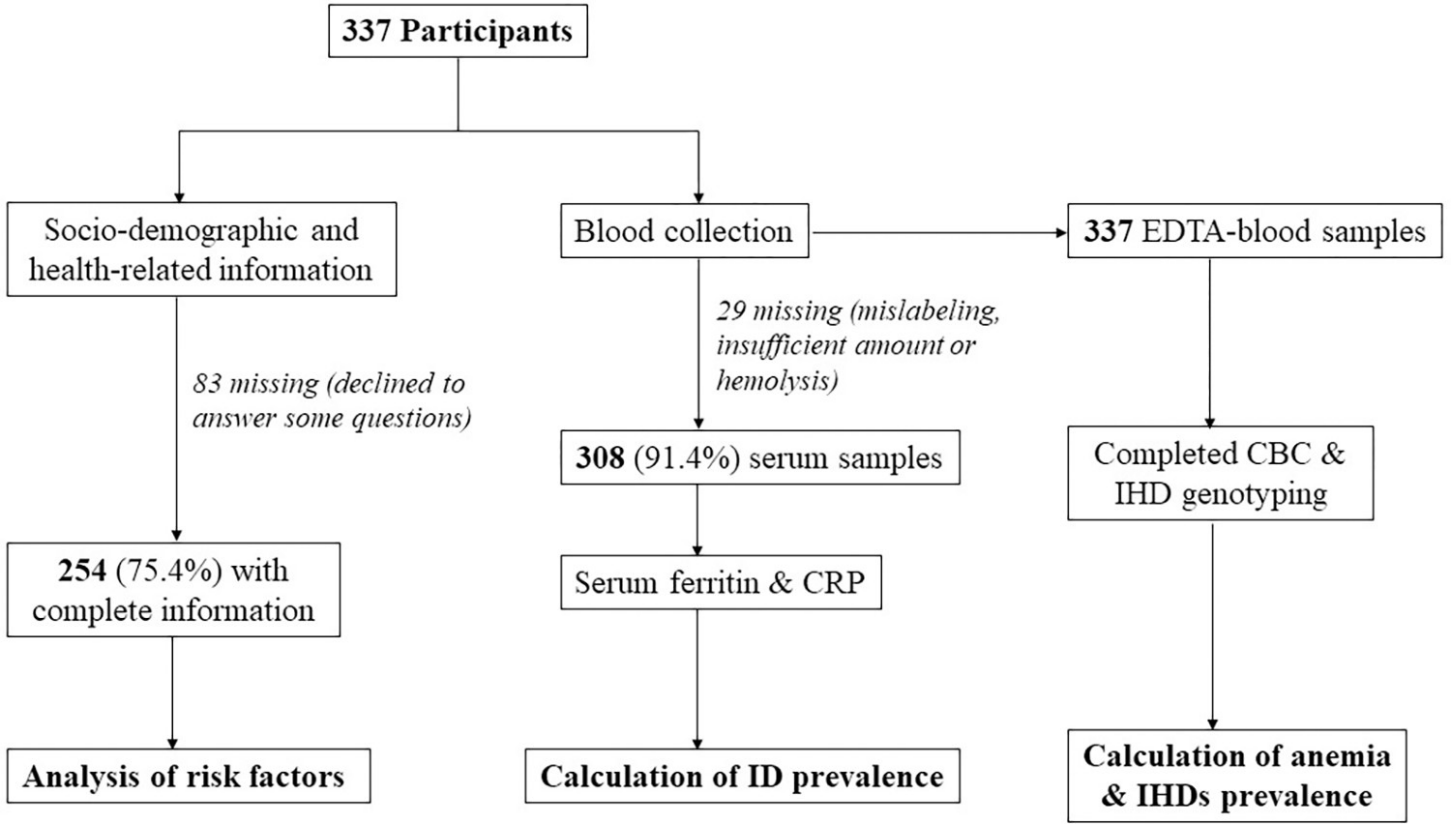
A flow diagram presenting data collection and number of samples eligible for statistical analysis.

Outcomes of the primary objective included anemia, IHDs and ID. The prevalence was expressed as percentage with 95% confidence interval (CI). The prevalence of anemia and IHDs were analyzed among 337 participants. For ID, the prevalence was calculated among 308 individuals in whom SF levels were available. A flow diagram presenting data collection and number of samples eligible for statistical analysis is shown in **Fig 1**.

To describe hematologic features, data distributions were initially tested by the Shapiro-Wilk test. Due to the non-normal distribution, data were presented as medians with interquartile range (IQR). Multiple comparison among groups was done using the Kruskal-Wallis test followed by the Conover post-hoc pairwise analysis. If appropriate, raw data were given.

Multiple logistic regression was applied to identify risk factors associated with moderate-to-severe anemia. After excluding cases with thalassemia diseases (i.e., 2 with Hb H and 2 with Hb E-β-thal diseases), the analysis was performed in 254 individuals who had complete sociodemographic information. Explanatory variables included IHD genotypes, ID, age group, sex, body mass index (BMI), educational level, occupation, underlying disease, and a family history of thalassemia disease. IHD genotypes were re-categorized into 5 groups; i.e., 1) non-IHD (reference group), 2) one α-gene defect (including heterozygous α^+^-thal and heterozygous Hb CS), 3) two α-gene defects (including heterozygous α^0^-thal, homozygous α^+^-thal, and compound heterozygous α^+^-thal with Hb CS), 4) β-thal carriers (with or without α-thal), and 5) Hb E carriers (with or without α-thal). Preselection of explanatory variables was performed using either the Chi-squared test or the Fisher’s exact test. Variables showing *p-value <0*.*25* were included in the multiple logistic regression model. Either the MedCalc® Statistical Software version 20.015 (MedCalc Software bvba, Ostend, Belgium; https://www.medcalc.org; 2021) or the Stata software version 12 (Stata Corp, Texas, USA) was used to analyze data. Statistical significance was set at *p-value < 0*.*05*.

## Results

Out of the 337 participants, 202 (59.9%) were females and 135 (40.1%) were males. The mean age was 43 years (SD = 15.6), and 63.8% were in the age of 18–49 years, with the prospect of having a child. According to BMI, 8.9% were underweight, 35.7% normal, 35.3% overweight and 20.1% obese. The majority of the participants (84.1%) were farmers. Eight persons (3.1%) held a bachelor’s degree, 39.5% finished high school or vocational school, and 36.8% went to primary school. Only 20.5% had been classified as illiterate. Approximately 16% had suffered or were suffering from a disease, including hypertension, asthma, diabetes, thyroid, gout, allergy, cataract and bone ailment. Approximately 6% remembered a family history of thalassemia. The general characteristics of the study participants are summarized in **Table 1**.

**Table 1:** General characteristics of the study participants.

Characteristic	Group category	n	(%)
Sex	Male	135	40.1
	Female	202	59.9
Age group (years)	18–49 (reproductive)	215	63.8
	50–65 (senior)	93	27.6
	> 65 (elderly)	29	8.6
BMI (kg/m^2^)^a^^,^^b^	< 18.5 (underweight)	23	8.9
	18.5–22.9 (Normal)	92	35.7
	23.0–27.5 (Overweight)	91	35.3
	>27.5 (Obese)	52	20.1
Educational level^a^	Illiterate	53	20.5
	Primary school	95	36.8
	High school or vocational school	102	39.5
	Bachelor’s degree or higher	8	3.1
Occupation^a^	Farmer	217	84.1
	Housewife/unemployed	20	7.7
	Business owner/private worker	11	4.3
	Others^c^	10	3.9
Underlying disease ^a^	No	217	84.1
	Yes	41	15.9
Major blood loss in the past 3 months (via accident or surgery)	No	255	98.8
	Yes	3	1.2
Currently taking iron supplementation	No	255	98.8
	Yes	3	1.2
Family history of thalassemia disease^a^	No	242	93.8
	Yes	16	6.2

a: Data were available in 258/337.

b: Categorized according to the BMI criteria for Asians [23]

c: Including student and government service

The anemia prevalence by severity and the overall frequencies of IHDs and ID among the study participants are summarized in **Table 2**. The prevalence of overall anemia was 27.9% (95% CI = 23.2–33.0). Mild anemia was detected in 18.7% of the total participants. Almost 10% of study participants had moderate anemia and <1% had severe anemia. Various forms of IHDs were identified in 166 participants, constituting 49.3% (95% CI = 43.8–54.7). Of the 308 participants, ID was detected in only 21 cases (6.8%, 95% CI = 4.3–10.2).

**Table 2:** Anemia prevalence by severity and the overall frequencies of IHDs and ID among the study participants.

Disorder	n	%	95% CI
Overall anemia	94	27.9	23.2–33.0
Mild^a^	63	18.7	14.7–23.3
Moderate^b^	30	8.9	6.1–12.5
Severe^c^	1	0.3	0.01–1.6
Overall IHDs	166	49.3	43.8–54.7
ID	21	6.8^d^	4.3–10.2

a: Hb = 110–119 g/L for females and 110–129 g/L for males

b: Hb = 80–109 g/L

c: Hb < 80 g/L, d: The percentage was calculated among 308 study participants in whom SF levels were available.

**Fig 2:**
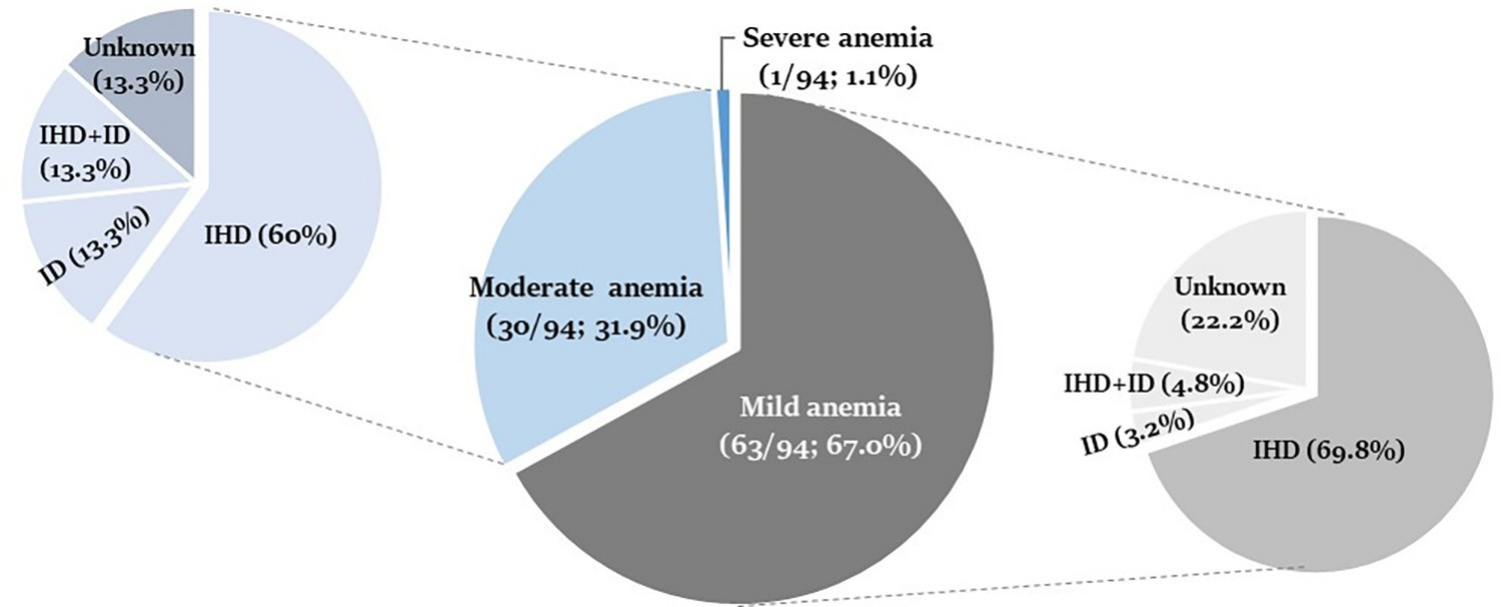
Explanatory factors among participants with mild and moderate anemia. Middle circle represents the proportion of anemia by severity; Left circle represents the proportions of IHD, ID, IHD+ID and unknown among individuals with moderate anemia; Right circle represents the proportions of IHD, ID, IHD+ID and unknown among individuals with mild anemia.

Explanatory factors among participants with mild and moderate anemia are shown in **Fig 2**. Of the 63 participants with mild anemia, 44 (69.8%) had IHDs. ID and the concurrence of IHD and ID (IHD+ID) were found in 2 (3.2%) and 3 (4.8%) participants, respectively. For moderate anemia, IHDs were detected in 18/30, constituting 60%. In this group, ID and IHD+ID were found in 4 persons each (13.3%). Anemia of unknown causes was found in 14 (22.2%) of mild anemia and 4 (13.3%) of moderate anemia. A case with severe anemia was found to be a β-thal carrier.

IHD genotypes among anemic and non-anemic participants are shown in **Table 3**. Heterozygous α^+^-thal (3.7 kb del) was most common (26.1%), followed by heterozygous states for β-thal (8.3% β^0^-thal and 0.3% β^+^-thal). The most common mutation causing β^0^-thal was IVS1-1 (G-T), followed by 3.4 kb deletion. Other β^0^-thal mutations included codon 17 (A-T) and codon 41/42 (-TTCT). The only mutation causing β^+^-thal identified among β-thal carriers was nucleotide -31 (A-G). Heterozygous states for α^0^-thal (SEA deletion) and Hb E were detected at relatively lower frequencies of 3.6% and 2.7%, respectively. Frequencies of homozygous states were 2.1% for α^+^-thal and 0.3% for Hb E. A low frequency of 0.6% was found for heterozygous Hb CS. For the complex forms, heterozygous α^+^-thal co-inherited with heterozygous β-thal was found commonly with a frequency of 2.7%. Heterozygous α^+^-thal co-inherited with heterozygous Hb E and compound heterozygous α^+^-thal/α^0^-thal (the Hb H disease) were identified at an equal frequency of 1.2%. Of the 166 IHD cases, mild and moderate severe anemia were detected in 47 and 22 persons, respectively. Comparing different genotypes, lower proportions of anemic cases were observed in heterozygous states for α^+^-thal and Hb E; and most of them were of mild severity. Of the 22 IHD cases exhibiting moderate anemia, 13 were found to be carriers of β-thal.

**Table 3:** IHD genotypes among anemic and non-anemic participants.

Genotype	Total (%)	No. of anemic cases			No. of non-anemic cases
		Mild	Moderate	Severe	
**α-thalassemia**					
Heterozygous α^+^-thal	88 (26.1)	15	5	0	68
Heterozygous α^0^-thal	12 (3.6)	6	1	0	5
Homozygous α^+^-thal	7 (2.1)	2	1	0	4
**β-thalassemia**					
Heterozygous β^0^-thal*	28 (8.3)	16	9	1	2
Heterozygous β^+^-thal*	1 (0.3)	1	0	0	0
**Structural Hb variants**					
Heterozygous Hb E	9 (2.7)	1	0	0	8
Heterozygous Hb CS	2 (0.6)	1	0	0	1
Homozygous Hb E	1 (0.3)	1	0	0	0
**Complex interaction of IHD**					
Het. β-thal with het. α^+^-thal	9 (2.7)	3	2	0	4
Het. Hb E with het. α^+^-thal	3 (0.9)	0	0	0	3
Compound heterozygous β^0^/β^+^-thal	2 (0.6)	1	1	0	0
Compound heterozygous α^0^-thal/α^+^-thal	2 (0.6)	0	2	0	0
Compound heterozygous α^+^ thal/Hb CS	1 (0.3)	0	0	0	1
Heterozygous β-thal with homozygous α^+^-thal	1 (0.3)	0	1	0	0
*Total IHD cases*	*166 (49*.*3)*	*47*	*22*	*1*	*96*
Non-IHD	171 (50.7)	16	8	0	147
**Total participants**	337 (100)	63	30	1	243

*Based on mutation analysis

**Table 4** illustrates hematologic features of ID and non-ID participants with different IHD genotypes. Compared to non-ID, individuals with ID had lower Hb, MCV and MCH values. Non-ID individuals with all forms of IHDs resulted in a significant reduction in Hb, MCV and MCH values but significant increase in RBC, as compared to the normal group (non-IHD/non-ID) of the same sex. Comparing between heterozygous states of IHDs, the lowest Hb, MCV and MCH values were observed in a group of heterozygous β-thal.

**Table 4:** Hematologic features of ID and non-ID cases with different IHD genotypes. The numbers in the table are median & IQRs.

Iron status	IHD genotype	Sex (n)	Hb (g/L)	RBC (x10^12^/L)	MCV (fL)	MCH (pg)
ID	non-IHD	F (10)	113 (104–123)*	4.5 (4.1–4.7)	82.7 (76.6–86.9)*	26.2 (23.9–27.2)*
		M (2)	114, 135	5.0, 5.1	73.0, 82.3	22.7, 26.2
	Het. α^+^-thal	F (3)	109 (91–115)	4.47 (4.4–4.51)	81.0 (68.5–82.1)	25.0 (20.1–25.7)
		M (1)	120	4.8	78.7	25.0
	Het. α^0^-thal	F (1)	110	5.0	71.9	22.2
	Homo. α^+^-thal	M (1)	113	6.4	61.8	17.8
	Het. β-thal	F (1)	112	6.0	61.3	18.8
		M (1)	136	7.0	62.6	19.3
	Het. β-thal with homo. α^+^-thal	F (1)	105	4.9	68.0	21.4
Non-ID	**α-thal**					
	Het. α^+^-thal	F (45)	126 (119–134)*	4.6 (4.4–4.9)*	86.7 (83.4–88.0)*	27.5 (26.9–28.2)*
		M (29)	140 (135–152)*	5.2 (4.9–5.5)*	85.8 (83.6–87.8)*	27.5 (26.8–28.0)*
	Het. α^0^-thal	F (7)	120 (114–121)*	5.3 (5.2–5.8)*	70.0 (67.3–71.9)*	21.5 (20.8–21.8)*
		M (3)	120 (118–127)	5.7 (5.3–6.0)	68.6 (67.5–74.0)	21.2 (20.9–22.4)
	Homo. α^+^-thal	F (5)	120 (108–129)	5.6 (4.8–5.7)	72.5 (70.0–74.1)	22.2 (21.8–22.9)
		M (1)	135	6.0	72.8	22.5
	**β-thal**					
	Het. β-thal	F (12)	108 (98–113)*	5.3 (4.2–5.5)*	67.5 (63.3–68.6)*	21.0 (19.6–22.7)*
		M (13)	118 (114–125)*	5.9 (5.6–6.3)*	65.7 (62.6–72.6)*	20.8 (19.5–21.2)*
	**Structural Hb variant**					
	Het. Hb E	F (7)	132 (128–134)	5.2 (4.8–5.3)*	77.3 (76.5–80.4)*	25.5 (24.8–26.0)*
		M (1)	154	5.5	85.6	28.0
	Het. Hb CS	F (1)	117	4.7	81.3	24.8
		M (1)	141	5.3	82.4	26.4
	Homo. E	M (1)	119	5.8	62.3	20.6
	**Complex interaction**					
	Het. β-thal with het. α^+^-thal	F (3)	114 (107–126)	5.2 (5.1–5.7)	69.8 (67.9–69.8)	22.0 (20.5–22.1)
		M (5)	133 (120–138)	5.8 (5.1–6.3)	71.8 (69.5–76.3)	22.8 (21.8–24.2)
	Het. Hb E with het. α^+^-thal	F (2)	144, 129	5.7, 5.1	78.0, 79.0	25.4, 25.5
	Comp. het. α^0^-thal/α^+^-thal	F (1)	89	5.4	56.2	16.5
	Comp. het. α^+^-thal/Hb CS	M (1)	134	5.4	81.2	24.9
	Comp. het. β^0^/β^+^-thal	F (1)^c^	91	4.4	67.1	20.8
		M (1)^d^	127	5.2	75.8	24.6
**Normal (non-ID/non-IHD)**		F (88)	132 (126–137)	4.4 (4.2–4.6)	92.0 (88.9–93.9)	30.0 (29.0–30.7)
		M (59)	145 (137–149)	4.7 (4.4–5.1)	93.2 (88.9–96.5)	30.5 (29.4–31.5)

Het.: Heterozygous; Homo.: Homozygous; Comp. het.: Compound heterozygous; F: female; M: male

*Statistically significant difference as compared to the normal (non-ID/non-IHD) group of the same sex

Preselection of factors associated with moderate-to-severe anemia revealed 6 candidate explanatory variables, including IHDs, ID, age, BMI, occupation and history of thalassemia. No association was observed for sex, education level and chronic disease. Applying multiple logistic regression, only 3 factors were found to be associated significantly with moderate-to-severe anemia, including β-thal, ID and age > 65 years. As shown in Table 5, while individuals heterozygous for β-thal were 6.2 times more likely to develop moderate-to-severe anemia than non-IHDs, the risk of individuals with ID were 17 times that of non-ID. For those aged > 65 years, the odds of having moderate-to-severe anemia were 8.1 times compared to the reproductive age group.

**Table 5:** Multiple logistic regression analysis of factors associated with moderate-to-severe anemia.

Factor	% Moderate-to-severe anemia (n)	Crude OR	Adjusted OR	95% CI	*p-value*
**IHD type**					
Non-IHD	3.6 (5/139)	1 (Reference)	1 (Reference)		-
One α-gene defect^a^	4.9 (3/61)	1.4	2.0	0.4–9.5	*0*.*386*
Two α-gene defects^b^	13.3 (2/15)	4.1	1.6	0.1–17.3	*0*.*697*
β-thal^c^	17.9 (5/28)	5.8	6.2	1.4–27.8	*0*.*016*
**Iron status**					
Non-ID	4.2 (10/238)	1 (Reference)	1 (Reference)		*-*
ID	31.3 (5/16)	10.4	17.0	3.8–75.2	*<0*.*001*
**Age group**					
18–49 years	3.6 (6/165)	1 (Reference)	1 (Reference)		-
50–65 years	5.7 (4/70)	1.6	2.4	0.6–10.2	*0*.*235*
> 65 years	26.3 (5/19)	9.5	8.1	1.6–40.4	*0*.*010*

a: Including heterozygous states for α^+^-thal and Hb CS

b: Including heterozygous α^0^-thal, homozygous α^+^-thal, and compound heterozygous α^+^-thal/Hb CS

c: Including heterozygous β-thal and those co-inherited with α^+^-thal

## Discussion

This study determined the burden of anemia and investigated IHDs molecularly among the Pwo-Karen ethnic minorities living in Ban Rai district, Uthai Thani province, Thailand. According to the village leader, poverty and inaccessibility to healthcare services remain the major problems of the community, and the burdens of anemia, IHDs and ID are not known. Our results show that the prevalence of anemia and IHDs was high, but the prevalence of ID was low. Although mild anemia was the most common, moderate and severe anemia were detected in approximately one-third of anemic participants. Further investigation of risk factors of moderate-to-severe anemia revealed that individuals with ID, β-thal and at the age > 65 years were at high risk.

The prevalence of anemia can vary from population to population depending on socio-demographic characteristics and ethnic background. Regardless of ethnicity, community-based studies in pregnant and non-pregnant women in low- and middle-income countries reported a wide range of anemia prevalence from 20–46% [4–6, 24–28]. While ID was found to be the main factor explaining anemia in some studies [24–27], others reported that IHDs were the major contributing factors [4–6, 28]. In this study, anemia prevalence of 27.9% among the Pwo-Karen people was comparable to previous studies conducted in Northeastern-Thai females of reproductive age [4–6, 28]. Due to the high frequency, higher proportions of IHDs were detected among anemic participants, and this indicates that IHDs should be taken into account when determining the anemia burden in this region. With regard to ID, anemia due to ID is expected to be high among poor indigenous people [29]. The finding of 6.8% ID among the Pwo-Karen people is therefore unexpected; and this could possibly be explained by the daily consumption of traditional foods that contain iron-rich vegetables and lime juice, with meats on occasion. These findings emphasize the requirement for a context-specific prevention of anemia.

In line with the global estimates for anemia [1], the majority of anemic cases were of mild severity. Although IHDs were found to be the main explanatory factors among anemic participants, a significantly higher proportion of ID (including a co-incidence of IHD and ID) was observed in a group with moderate anemia (Fig 2), suggesting that ID could seriously affect individual health. Given the fact that both iron and globin protein are the main components of the Hb molecule, ID and IHDs could affect Hb production and RBC morphology directly. As supported by the data shown in Table 4, individuals with either ID or IHDs tended to have lower Hb concentration as well as MCV and MCH values as compared to non-ID and non-IHDs. With ID, anemia in IHD carriers was more pronounced. These hematologic changes are consistent with data demonstrated in previous reports [4, 26]. Given that IHDs are caused by gene abnormalities, no treatment is required for a heterozygous state with mild anemia. Unlike IHDs, ID individuals require iron supplementation in order to prevent the progression of anemia. It is therefore crucial for healthcare staff to understand the causative factors related to anemia in each community, so that they can provide appropriate management and care for the affected individuals.

It should be noted that not all forms of IHDs resulted in anemia. Without ID, heterozygous states for α^+^-thal and Hb E appear to have no effect on anemia, whereas other forms of IHDs (including β-thal, α^0^-thal, 2 α-globin gene defects, and the complex interaction of IHDs) resulted in microcytic anemia with varying severities. Detailed analysis of IHD genotypes in relation to anemia severity reveals that moderate anemia was found mainly in heterozygous β-thal and its interaction with other forms of IHDs (Table 3), suggesting that β-thal may have a stronger effect on anemia than other heterozygous states. Further analysis for factors associated with moderate-to-severe anemia confirms that ID and β-thal individuals were at higher risk than the respective reference groups. Of note, while the effect of ID on anemia was consistent with previous studies, the type of IHD that contributed to anemia was not similar. Unlike this study, previous reports in the northeast-Thai population have demonstrated that two α-gene defects (including α^0^-thal) and Hb E were the major determinants of anemia [4, 6, 28], and this could be explained by the different ethnic background.

In addition to IHDs and ID, there remains unknown causes among anemic participants. Deficiencies in other micronutrients, such as vitamin B12 and folate, could possibly have contributed to anemia among those with unknown causes. Further studies are needed to confirm such a possibility. However, it is evident in this study that age > 65 years was associated significantly with moderate-to-severe anemia, indicating that the aging process could aggravate anemia and might contribute in part to anemia of unknown causes. With increasing age, a decreased erythropoietic activity has been demonstrated [30–33]. While aging with some forms of IHDs, anemia could worsen.

Little is known about the molecular basis of IHDs among ethnic minority groups in Thailand. Previous studies at the north and northwestern border of Thailand reported the overall frequency of 15.6% for Hb E, β-thal and Hb CS in pregnant women of ethnic minorities [34], and 23.4% for α-thal among the Tai and Mon-Khmer ethnic groups [35]. Another population-based screening among six different hill tribes in northern Thailand resulted in a positive thalassemia test of 9.8% [36]. In this study, the overall frequency of IHDs was much higher. In particular, a surprisingly high frequency of β-thal indicates that the burden of severe thalassemia syndrome due to homozygous β-thal might be serious. In addition, the detections of 4.2% α^0^-thal and 3.9% Hb E reflect the high probability of having newborns with homozygous α^0^-thal (so-called Hb Bart’s hydrops fetalis) and Hb E-β-thal diseases. While babies born with Hb Bart’s hydrops fetalis usually die shortly after birth, babies born with homozygous β-thal and Hb E-β-thal diseases may develop severe anemia at 1–2 years of age [4]. Although severity of anemia may vary from patient to patient, all severe cases require regular blood transfusion; and this causes not only a poor quality of life but also an economic burden to the affected families.

Considering that this study was a community-based cross-sectional study conducted in a homogenous group of ethnic minorities, some limitations should be borne in mind. Firstly, as we included participants from all age ranges from 18–87 years, the findings of anemia prevalence and its risk factors may not be generalizable to any specific age range. Secondly, incomplete socioeconomic information and the loss of some serum samples led to a smaller sample size for risk factors analysis. Nonetheless, this does not affect the analysis of anemia prevalence and IHDs. Thirdly, other causes of anemia, which might contribute in part to anemia, were not investigated. Fourthly, as the Karen being studied are of the Pwo subgroup, types and frequencies of IHDs may not represent other subgroups of the Karen people. In addition, since we applied convenience sampling to recruit participants, an individual in the same family might be included in the study, and this could affect the estimation of IHD prevalence to some extent.

## Conclusions

This study reports for the first time the burdens of anemia, ID and IHDs among the Pwo-Karen residing in lower-northern Thailand. Anemia prevalence in this Karen community is of moderate public health significance in which IHDs are the major factors explaining anemia. Because of the high risk of developing moderate-to-severe anemia, special attention should be paid to individuals affected with ID and β-thal as well as the elderly. Responsible health staff should be trained to be aware of the genetic background for development of moderate and/or severe anemia. The population also should be enlightened and projects like the one for thalassemia prevention within the Thai population should be considered for adjustment to the minority groups. The mutations of α- and β-globin genes reported herein will be a good reference for further studies on population genetics in the region.

## Supporting information

S1 Data (XLSX)

S1 Table Proportions of anemia, IHDs, and ID in relation to sex, age and BMI groups. (DOCX)

S2 Table Anemia severity categorized by age groups and IHD status. (DOCX)
