# Supplementary material for: Anemia in an ethnic minority group in lower northern Thailand: A community-based study investigating the prevalence in relation to inherited hemoglobin disorders and iron deficiency
Source: PLoS One. 2023 Jun 23;18(6):e0287527. doi: 10.1371/journal.pone.0287527 (PMC10289360; doi:10.1371/journal.pone.0287527)
Supplement: S1 Table — (DOCX) [file pone.0287527.s002.docx]

**Table S1** Proportions of anemia, IHDs, and ID in relation to sex, age and BMI groups

| Characteristic | Group category | %Anemia | %IHDs | %ID* |
| --- | --- | --- | --- | --- |
| Sex | Male | 28.2 (38/135) | 50.4 (68/135) | 4.2 (5/120) |
|  | Female | 27.7 (56/202) | 48.5 (98/202) | 8.5 (16/188) |
| Age group (years) | 18-49 (reproductive) | 25.6 (55/215) | 47.0 (114/215) | 7.7 (15/194) |
|  | 50-65 (senior) | 22.6 (21/93) | 38.7 (36/93) | 2.3 (2/87) |
|  | > 65 (elderly) | 62.1 (18/29) | 55.2 (16/29) | 14.8 (4/27) |
| BMI (kg/m^2^)^a,b^ | < 18.5 (underweight) | 52.2 (12/23) | 56.5 (13/23) | 10.5 (2/19) |
|  | 18.5-22.9 (Normal) | 29.4 (27/92) | 47.8 (44/92) | 6.4 (5/78) |
|  | 23.0-27.5 (Overweight) | 16.5 (15/91) | 41.8 (38/91) | 7.1 (6/84) |
|  | >27.5 (Obese) | 15.4 (8/52) | 46.2 (24/52) | 4.2 (2/48) |

*Data were available in 308 samples.
