# Supplementary material for: Anemia in an ethnic minority group in lower northern Thailand: A community-based study investigating the prevalence in relation to inherited hemoglobin disorders and iron deficiency
Source: PLoS One. 2023 Jun 23;18(6):e0287527. doi: 10.1371/journal.pone.0287527 (PMC10289360; doi:10.1371/journal.pone.0287527)
Supplement: S2 Table — (DOCX) [file pone.0287527.s003.docx]

**Table S2** Anemia severity categorized by age groups and IHD status

| Age group (N) | IHD status | No. of cases with anemia (%) | | Non anemia |
| --- | --- | --- | --- | --- |
|  |  | Mild | Moderate-to-severe |  |
| 18-49 years (215) | Non-IHD (101) | 7 (6.9) | 5 (5.0) | 89 (88.1) |
|  | With IHD (114) | 31 (27.2) | 12 (10.5) | 71 (62.3) |
| 50-65 years (93) | Non-IHD (57) | 6 (10.5) | 1 (1.8) | 50 (87.7) |
|  | With IHD (36) | 9 (25.0) | 5 (13.9) | 22 (61.1) |
| > 65 years (29) | Non-IHD (13) | 3 (23.1) | 2 (15.4) | 8 (61.5) |
|  | With IHD (16) | 7 (43.75) | 6 (37.5) | 3 (18.75) |
